# Treatment Gaps Found in the Management of Type 2 Diabetes at a Community Health Centre in Johannesburg, South Africa

**DOI:** 10.1155/2017/9536025

**Published:** 2017-10-10

**Authors:** Yacob Pinchevsky, Neil Butkow, Tobias Chirwa, Frederick Raal

**Affiliations:** ^1^Department of Pharmacy and Pharmacology, School of Therapeutic Sciences, Faculty of Health Sciences, University of the Witwatersrand, Johannesburg, South Africa; ^2^Division of Epidemiology and Biostatistics, School of Public Health, Faculty of Health Sciences, University of the Witwatersrand, Johannesburg, South Africa; ^3^Carbohydrate and Lipid Metabolism Research Unit, Division of Endocrinology and Metabolism, Faculty of Health Sciences, University of the Witwatersrand, Johannesburg, South Africa

## Abstract

**Aims:**

The management of cardiometabolic goals or “ABCs” (HbA1c, blood pressure (BP), and cholesterol) ultimately determines the morbidity and mortality outcomes in patients with type 2 diabetes mellitus (T2DM). We sought to determine if patients with T2DM attending an urbanized public sector community health centre (CHC) were having their ABCs measured, were treated with appropriate cardioprotective agents and finally, were achieving guideline-based targets.

**Methods and Results:**

A cross-sectional record review of 519 patients was conducted between May and August 2015. The mean age was 54 years (SD: ±11.5) and 54% (*n* = 280) were females. Testing of ABCs occurred in 68.8% (*n* = 357) for HbA1c, 95.4% (*n* = 495) for BP, and 58.6% (*n* = 304) for LDL-C. Achievement of ABC targets was as follows: 19.3% (HbA1c < 7%), 22.0% (BP < 140/80 mmHg), and 56.3% (LDL-C < 2.5 mmol/l).

**Conclusion:**

There were a significant number of patients who were not tested nor received adequate pharmacotherapy or achieved their ABC targets. This places these patients at an increased risk for the development of diabetes-related complications. Although the realities of resource constraints exist in South Africa's public sector settings, a wider implementation of evidence-based guidelines must be instituted in order to ensure better patient outcomes.

## 1. Introduction

Diabetes mellitus (DM) is a chronic-progressive, multifactorial condition leading to a host of serious complications. In 2015, it was estimated that 415 million people had diabetes. However, this figure is expected to rise to 642 million in the next 25 years [1]. The DM condition has earned itself a place among the 10 leading causes of death worldwide [2]. Landmark studies such as the Diabetes Control and Complications Trial (DCCT) and the United Kingdom Prospective Diabetes Study (UKPDS) have shown that improved glycaemic control resulted in lowered micro- and/or macrovascular complication rates [3, 4]. Thus, screening and control of risk factors that are typically associated with DM such as hyperglycaemia, raised blood pressure (BP), and dyslipidaemia have been incorporated into evidence-based guidelines [5]. Yet, achievement of the abovementioned risk factors to target levels remains elusive in clinical practice.

As the rates of DM continue to rise, so too do concerns of the abilities of a healthcare system to deliver quality healthcare. Especially in lower resource settings such as those found in South Africa, the DM condition and its associated complications challenge healthcare systems. Therefore, using disease-specific indicators to measure the preventive treatment prescribed and quality of diabetes care available, we sought to benchmark a healthcare setting's performance [6]. For this study, we aimed to comprehensively evaluate the diabetes processes of care, pharmacological treatments prescribed (ACE inhibitors/metformin/statins), and achievement of intermediate outcomes of ABCs (HbA1c/BP/cholesterol) in a South African adult population with type 2 diabetes mellitus (T2DM) attending an urban public sector community health centre (CHC).

## 2. Material and Methods

Our study took place at the Hillbrow CHC between May and August 2015. This chosen setting is an academic provincial secondary level healthcare facility associated with the University of the Witwatersrand, Johannesburg, South Africa. The clinic offers emergency services, minor theatre procedures, polyclinic, outpatient services, psychology, and dental care [7].

The cohort size consisted of 519 patients with previously diagnosed T2DM. In our study, T2DM was defined as patients >18 years of age and/or had “T2DM” written in their records and/or having evidence of a prescription which featured an oral hypoglycaemic/insulin. Patients who met one or more of the inclusion criteria of “having a positive diagnosis for T2DM” as above were included in the study. All of the data for this study was retrospectively abstracted from patient records. Patient's files were chosen in a consecutive manner based on appointments for the day, until the sample size needed was met. Data abstracted from patient records included the following: demographics, medical history, laboratory results, and pharmacological treatment prescribed.

During the data collection period (May to August 2015), only the latest medical information was abstracted from the patient records. In the case where data was found to be missing from the latest records, we chose to include information from patient records as far back as 1 year from the time data collection commenced (i.e., May 2014). The blood pressure measurements were assumed to have been conducted by nurses or doctors at the clinic and in line with the South African hypertension Guidelines [8]. Standard procedures were also assumed to have been followed when blood was drawn from patients. The HbA1c was determined using the Tina-quant Haemoglobin A1c II immunological assay. Using the Modular Analyser P800 System (Roche Diagnostics-Hitachi, Mannheim, Germany), the direct and colorimetric enzymatic methods were utilised for determining the total cholesterol (TC), high-density lipoprotein cholesterol (HDL-C), and triglycerides, respectively. The low-density lipoprotein cholesterol (LDL-C) was calculated from the Friedewald equation. Patients prescribed antihypertensive treatment were assumed to have been hypertensive.

For the purposes of this retrospective study, macrovascular disease was considered present in patients who were previously diagnosed (as per their medical record) with any one of the following: cardiovascular disease, ischaemic heart disease, coronary artery disease, stroke, or transient ischaemic attack. Guidelines recommend the detection of microvascular complications through eye examinations (to detect retinopathy), feet inspections (neuropathy), and microalbuminuria testing (nephropathy) [5]. For our study, microvascular disease was only reported if any one of the following complications were recorded in patient records: retinopathy, neuropathy, or nephropathy.

The target levels applied to each risk factor was obtained from the Society for Endocrinology, Metabolism and Diabetes of South Africa (SEMDSA) 2012 Guidelines, which was the latest at the time of the study. This included the following targets: HbA1c < 7.0%, systolic BP < 140 mmHg, diastolic BP < 80 mmHg, TC < 4.5 mmol/l, LDL-C < 2.5 mmol/l, HDL-C > 1.0 mmol/l (men), HDL-C > 1.2 mmol/l (women), and triglyceride < 1.7 mmol/l.

Patient records at the clinic lacked weight and height detail which unfortunately meant body mass index (BMI) calculations were not possible. Waist circumference and smoking status data were also poorly recorded and therefore excluded from the study. Our sample size calculations were based on the HbA1c, BP, and LDL-C measurements which we assumed would be similar to those levels found in a previous local T2DM population-based study in a nearby hospital [9]. The confidence levels selected were 95% (significance level of 5%) and a power of 80% was chosen. The largest number obtained from calculations of the three intermediate outcomes (HbA1c, systolic BP, and LDL-C) was used to calculate the sample size chosen for the study. Descriptive analyses of summary measures were calculated for demographic data, HbA1c, BP, and lipids. The Student's *t*-test was used to explore key continuous measurements. Frequency tables for patient usage of chronic treatment used in glycaemia, hypertension, and lipid-lowering were produced. The % of patients achieving SEMDSA treatment targets for different clinical parameters was calculated. Microsoft Office Excel 2010 (Microsoft, Redmond, WA, USA) and Statistica version 13 (64-bit) (Dell Inc. Tulsa, OK 74104, USA) were used for the database and analysis. The University of the Witwatersrand's Human Research Ethics Committee approved the study prior to commencement.

## 3. Results

There were a total of 519 patients whose records we retrospectively reviewed in this study. Over half the sample (53.9%) were female, and the mean age for the group was 53.9 years (SD: 11.5). All but four patients were of African descent (Table 1).

In the study cohort, 162 (31.2%) of patients did not have HbA1c levels readily available in their records. Patients with HbA1c levels had a mean HbA1c of 9.1% (±2.6), a median of 8.5%, and 19.3% achieved target levels of <7% (Tables 1 and 2). In comparison with the hypoglycaemic treatment strategies available, the majority of patients (38.9%) were found to have been prescribed a combination of one oral hypoglycaemic agent together with insulin (Table 1). Monotherapy oral hypoglycaemic agents were prescribed to 115 (22.2%) whilst insulin was prescribed to 47 (9.1%), also as monotherapy. The HbA1c for patients using an oral agent as monotherapy was 8.7% (±2.6), which was lower than the HbA1c for those using monotherapy insulin 9.3% (±2.1) (*p* = 0.46). The patients using combinations of orals/insulin had an HbA1c of 9.5% (±2.6). Similarly, 25.9% of patients who were on orals (no insulin), 14.5% of patients on a combination of orals/insulin, and 10.7% of those using insulin (no orals) achieved target levels of HbA1c of <7% (*p* = 0.09). Of the few patients (*n* = 9) who were not treated on oral nor insulin, only 1 achieved HbA1c < 7%. The biguanides, in the form of metformin (the 850 mg strength only), were the most frequently prescribed agents amongst the total users of oral hypoglycaemic agents in the study, 450/463 (97.2%). Between the two types of sulphonylurea (SU) agents available to patients at this clinic, the majority (86.2%) utilised glimepiride (4 mg strength most frequently) whilst fewer (13.8%) were on gliclazide (160 mg strength most frequently).

Almost the entire cohort was treated for hypertension, *n* = 459/519 (88.4%). Only 24 (4.6%) patients had missing blood pressure measurements (Table 3). Those with levels available, their mean BP was 138/82 mmHg (Table 1). Just over one-half (52.7%) achieved SBP targets (<140 mmHg), whilst over one-quarter (28.7%) achieved DBP targets (<80 mmHg) (*p* < 0.01) (Table 2). The combined achievement of <140/80 mmHg occurred in 22.0%. Combinations of ≥2 antihypertensive agents were used by the majority of patients (74.9%) in comparison with monotherapy (25.1%) (*p* < 0.01). The average used 2.5 antihypertensive medications. Those not using antihypertensives had a mean BP of 126/78 mmHg versus 139/82 mmHg for patients on treatment (*p* < 0.01). The achievement of combined BP targets (<140/80 mmHg) by 1, 2, 3, or 4 combinations of antihypertensive classes was 22.3%, 22.3%, 18.3%, and 19.7%, respectively. ACE inhibitors were the most commonly utilised class of antihypertensives 403 (87.8%), followed by 300 (65.4%) users of diuretics, 262 (57.1%) users of calcium channel blockers and 89 (19.4%) on *β*-blockers.

Lipids level measurements were missing in just over 40% of patient records for all lipid categories (Table 3). Over half the cohort achieved TC (65.6%, <4.5 mmol/l), TG (64.0%, <1.7 mmol/l), LDL-C (56.3%, <2.5 mmol/l), and HDL-C targets (52.6%, >1 mmol/l in men and 23.7%, >1.2 mmol/l in women) (Table 2). Lipid-lowering treatments (statins) were prescribed to 133 (25.6%) patients. Of the patients who were not achieving the LDL-C < 2.5 mmol/l target, 41.7% were not prescribed statin treatment.

Simvastatin was the only statin available to patients at the clinic, and the 20 mg dosage was the most frequently prescribed in 122/133 (91.7%) of users. Fewer statin users achieved their LDL-C targets of <2.5 mmol/l (24.0%, mean LDL-C 1.83 ± 0.46) in comparison with those who were not using statins (76.0%, mean LDL-C 1.79 ± 0.41) (*p* = 0.23).

## 4. Discussion

This cross-sectional study set out to assess the frequency of testing and control of critical risk factors in a South African population of adult patients with T2DM attending a public sector CHC. The principal observation of this study was that a significant portion of patients had missing measurements and failed to achieve guideline-based targets. In particular, our main focus was to determine the management of ABCs (HbA1c, BP, and cholesterol) which ultimately determines the morbidity and mortality outcomes in T2DM patients. The majority (83%) of South Africans access their healthcare within the public sector and, therefore, our study provides a necessary and important means by which to demonstrate the quality of care available to patients within the state sector.

Evidence-based guidelines recommend that patients with T2DM be treated to an optimal HbA1c of <7%. Approximately two-thirds of our cohort (68.8%) had HbA1c measurements available. Disappointingly, only 1/5 of patients reached an HbA1c <7%. However, studies from other South African sites have also reported such unsatisfactory findings whereby as few as 11.2–15.6% achieved HbA1c < 7% [10, 11]. In contrast, some other local studies showed better results than what we found, albeit not necessarily by much: 27.0%–30.7% achieved HbA1c < 7% [12, 13]. Studies across Europe had as many as 53.6% of patients with HbA1c <7% (combined total of eight countries) [14]. This European data was also consistent with findings from a United States study where 52.5% of people with diabetes achieved HbA1c <7% [15].

Results from the UKPDS indicated that the ideal HbA1c levels were best achieved through combinations of oral and insulin therapies as the T2DM progressed [16]. With only 9 patients not using hypoglycaemic agents nor insulin, perhaps practitioners at this setting should be encouraged to initiate pharmacotherapy at an earlier stage of the disease. Our study may have also occurred at a time when many of the T2DM study patients did not appear to show improvements in their HbA1c levels through lifestyle modifications (i.e., diet, physical activity, and stress reductions). Almost half our population were on a combination of multiple therapies. Our “combination group” had worse HbA1c control in comparison with patients on oral therapy alone. However, control was in fact worse in the oral insulin combination group versus the insulin monotherapy group. Perhaps, this indicates that the subset of combination patients was only recently prescribed the additional insulin (on top of their oral agents) at the time of this study and their HbA1c was still to improve. Potentially, poor insulin injecting technique by patients or therapeutic inertia by practitioners in response to the eroded glycaemic control may have also been the reason.

Hypertension remains the most common life-threatening risk factor for cardiovascular disease in developing countries [17]. As many as 88.4% of our subjects were hypertensive, which is similar to other independent local studies. For instance, Webb et al. had >79% (Pretoria, South Africa), Klisiewicz found 85% (Johannesburg, South Africa), and there were 77.9% in the World Health Organization Study on Global Aging and Adult Health in South Africa [12, 13, 18]. Approximately one-quarter of the study population achieved both systolic and diastolic BP targets (<140/80 mmHg)—a figure that is relatively less successful than the ones reported in other studies [9, 10]. The poor BP control in our patients may be attributed to inadequate treatment. A quarter of the cohort was treated with monotherapy, and the rest were on an average of 2.5 antihypertensive medications. Patients with T2DM typically require 3-4 classes of antihypertensives in order to achieve BP targets [19, 20]. However, as per guideline recommendations, the use of ACE inhibitors was a widely accepted practice at our clinic. The high use of ACE inhibitors in nearly all hypertensives in the study may have occurred due to (1) formulary restrictions at public healthcare facilities, whereby newer and possibly more expensive angiotensin receptor blockers were not routinely available; (2) patients required a combination of antihypertensives treatments in order to achieve better control of their blood pressure; therefore, many patients were started on diuretics together with an ACE inhibitor; (3) practitioners opted for the use of ACE inhibitors in their patients due to its established renal protective benefits over other antihypertensive drug classes; and (4) calcium channel blockers may have only been prescribed when patients were not responsive to ACE inhibitors or experienced side effects from ACE inhibitors [21].

One of the key challenges associated with the treatment of hypertension is the asymptomatic and chronic nature of the condition [22]. Poor medication compliance due to side effects, complicated or frequent dosing, mindfulness of normal BP levels, and an inattentive community/healthcare system may also contribute to the poor achievement of BP targets [23]. Perhaps, patients had resistant hypertension or were only recently diagnosed with hypertension and were yet to stabilize on an antihypertension treatment.

The achievement of lipid targets in our study patients was superior to those of BP and HbA1c. One-half of patients in our study achieved LDL-C < 2.5 mmol/l as per the guideline recommendations. Our results demonstrate higher achievement rates of LDL-C targets in comparison with some other local [12, 13] and international studies [14, 24]. Perhaps, this may be linked to the fact that so few patients (<1%) attending this CHC had macrovascular disease which may suggest that the more “complicated variety” of T2DM patients was referred to a nearby Johannesburg tertiary level facility (where macrovascular rates appear as high as 22.2%) [25]. However, an astonishing 41.7% of our patients who were not achieving the LDL-C < 2.5 mmol/l target were also not receiving statin treatment. Statin usage appeared to be particularly low in our setting (25.6%) in comparison with other settings (77.8%) [25], which not only demonstrates poor guideline adherence but also provides reasons why more of our patients did not achieve guideline recommended LDL-C levels. Practitioners may have also been relying on alternate means such as diet or other lifestyle modifications in order to get patients to target. Previous studies have found that achievement of LDL-C targets is not only more easily achievable than HbA1c and BP, but its impact far outweighs the benefits [26]. Perhaps, practitioners at our CHC were too “glucocentric” in their approach to T2DM management.

Compelling evidence suggests that optimal vascular protection is achieved through a multifactorial approach (e.g., use of antiplatelet therapy, ACE inhibitors, metformin, and statins) thereby yielding risk reduction in patients with T2DM [27]. Only three patients (0.6%) at our clinic were noted to have had macrovascular disease and therefore it is plausible that acetylsalicylic acid may have been overprescribed to individuals (26.2% of our population received antiplatelet agents) who had not yet developed atherosclerotic disease. Perhaps, the low complication rates which we have reported are in fact indicative of insufficient and low numbers of screenings performed in patients at this setting.

Our study had several limitations. Our analyses were confined to only those patient records available at the time of the study. Although consistent, the latest guidelines at the time of this study were used, thereby applying less stringent targets and making more patients appear to achieve their targets (e.g., systolic BP targets of 140 mmHg when previously in 2009, 130 mmHg) [5]. The treatment and care received by patients at the Hillbrow CHC do not necessarily reflect the typical level of care provided across all CHCs. Some rural CHCs are nurse-run (no doctors/pharmacists), have more restrictive formularies, and may lack access to laboratory facilities. As this study took place in an urbanized setting with a select group of Black African patients who presented with T2DM, the generalizability of this study may not be entirely applicable to all diabetics across South Africa. Our setting is more reflective of care available at the urban secondary level. Patient records had missing data and therefore our study may have lacked details of diabetes duration, waist circumference, height, and smoking status. In addition, complication rates were found to be extremely low which may suggest an underreporting of complications through possible lack of screenings. The cross-sectional methodology carried out in this study only reflects the once off measurement levels of patients at the time of the study. Although it would have been beneficial to have studied larger patient numbers (as typically found in epidemiological studies which utilise massive electronic HMO databases); however, our setting was limited in that it did not feature any electronic systems and therefore each record was manually abstracted by hand in order to obtain the sample size needed for the study (see Materials and Methods).

The present study demonstrated that a large portion of patients had missing measurements and did not achieve guideline-based targets. Effective treatment and interventions can reduce risk in patients with T2DM. However, we found that a suboptimal level of preventative and therapeutic approaches was implemented at this setting. Improved risk factor testing and clinical inertia were some of the challenges observed, which have its ramifications in the quality of diabetes care delivered. Future interventions should focus on initiating more aggressive preventive strategies through an earlier use of combination therapy, addressing barriers to physician clinical inertia, or patient compliance to treatment.

## Figures and Tables

**Table 1:** Clinical characteristics: patients with T2DM attending the Hillbrow Community Health Centre.

Characteristic	Number of patients and % of those tested with variable
Age (years)	53.9 ± 11.5
Female sex, *n* (%)	280 (53.9)
Ethnicity: Black African, *n* (%)/other, *n* (%)	515 (99.2)/4 (0.8)
Cardiovascular disease, *n* (%)	2 (0.4)
Stroke/TIA, *n* (%)	1 (0.2)
Retinopathy, *n* (%)	4 (0.8)
Neuropathy, *n* (%)	3 (0.6)
Nephropathy, *n* (%)	1 (0.2)
HbA1c (%)	9.1 ± 2.6
Systolic BP (mmHg)	138 ± 18.4
Diastolic BP (mmHg)	82 ± 10.3
TC (mmol/L)	4.2 ± 1
TG (mmol/L)	1.5 ± 0.9
LDL-C (mmol/L)	2.4 ± 0.9
HDL-C (mmol/L)	1.1 ± 0.3
*Glycaemic control agents*	
Diet only, *n* (%)	9 (1.7)
1 oral, no insulin, *n* (%)	115 (22.2)
≥2 oral, no insulin, *n* (%)	140 (27.0)
Insulin only, *n* (%)	47 (9.1)
Combination—insulin with 1 oral, *n* (%)	202 (38.9)
Combination—insulin with ≥2 oral, *n* (%)	6 (1.2)
Biguanides, *n* (%)	450 (86.7)
Sulphonylureas, *n* (%)	159 (30.6)
*Antihypertensive agents*	
Antihypertensive treatment, *n* (%)	459 (88.4)
Monotherapy, *n* (%)	115 (25.1)
Combination therapy (2 classes), *n* (%)	121 (26.4)
Combination therapy (3 classes), *n* (%)	132 (28.8)
Combination therapy (4 classes), *n* (%)	67 (14.6)
Combination therapy (≥5 classes), *n* (%)	24 (5.2)
ACE inhibitors, *n* (%)	403 (87.8)
*Lipid-lowering agents*	
Statin treatment, *n* (%)	133 (25.6)
*Other agents*	
Antiplatelet treatment, *n* (%)	136 (26.2)
Thyroid treatment, *n* (%)	6 (1.2)

BP: blood pressure; HbA1c: glycated haemoglobin; HDL: high-density lipoprotein; LDL: low-density lipoprotein; TC: total cholesterol; TG: triglyceride; TIA: transient ischaemic attack.

**Table 2:** Intermediate outcomes: patients with T2DM attending the Hillbrow Community Health Centre.

Characteristic	Number of patients and % of those achieving target (no missing values)	Number of patients and % of those achieving target (total group)
HbA1c (<7%), *n* (%)	69 (19.3)	69 (13.3)
Systolic BP (<140 mmHg), *n* (%)	261 (52.7)	261 (50.3)
Diastolic BP (<80 mmHg), *n* (%)	142 (28.7)	142 (27.4)
Combined BP (<140/80 mmHg), *n* (%)	109 (22.0)	109 (21.0)
TC (<4.5 mmol/l), *n* (%)	202 (65.6)	202 (38.9)
TG (<1.7 mmol/l), *n* (%)	197 (64.0)	197 (38.0)
LDL-C (<2.5 mmol/l), *n* (%)	171 (56.3)	171 (32.9)
HDL-C (>1 mmol/l in men), *n* (%)	162 (52.6)	162 (31.2)
HDL-C (>1.2 mmol/l in women), *n* (%)	73 (23.7)	73 (14.1)

BP: blood pressure; HbA1c: glycated haemoglobin; HDL: high-density lipoprotein; LDL: low-density lipoprotein; TC: total cholesterol; TG: triglyceride.

**Table 3:** Processes of care: measurements taken in patients with T2DM attending the Hillbrow Community Health Centre.

Characteristic	Number of patients and % of those being tested for variable
HbA1c, *n* (%)	357 (68.8)
Systolic BP, *n* (%)	495 (95.4)
Diastolic BP, *n* (%)	495 (95.4)
TC, *n* (%)	308 (59.3)
TG, *n* (%)	308 (59.3)
LDL-C, *n* (%)	304 (58.6)
HDL-C, *n* (%)	308 (59.3)

BP: blood pressure; HbA1c: glycated haemoglobin; HDL: high-density lipoprotein; LDL: low-density lipoprotein; TC: total cholesterol; TG: triglyceride.
